# Evaluating the Impact of Bowel Gas Variations for Wilms’ Tumor in Pediatric Proton Therapy

**DOI:** 10.3390/cancers16030642

**Published:** 2024-02-02

**Authors:** Ozgur Ates, Fakhriddin Pirlepesov, Jinsoo Uh, Chia-ho Hua, Thomas E. Merchant, Andrew Boria, Andrew M. Davidoff, Dylan E. Graetz, Matthew J. Krasin

**Affiliations:** St. Jude Children’s Research Hospital, Memphis, TN 38105, USA; fakhriddin.pirlepesov@stjude.org (F.P.); jinsoo.uh@stjude.org (J.U.); chia-ho.hua@stjude.org (C.-h.H.); thomas.merchant@stjude.org (T.E.M.); andrew.boria@stjude.org (A.B.); andrew.davidoff@stjude.org (A.M.D.); dylan.graetz@stjude.org (D.E.G.); matthew.krasin@stjude.org (M.J.K.)

**Keywords:** Wilms’ tumor, pediatric proton therapy, bowel gas, water equivalent path length, WEPL, proton range uncertainty

## Abstract

**Simple Summary:**

The manuscript discusses the administration of proton therapy to the first 13 pediatric patients in the prospective SJWT21 clinical trial to assess the efficacy of proton therapy in treating children with stage III-V Wilms’ tumor. The group of 13 patients received abdominal irradiation after undergoing a nephrectomy or partial nephrectomy. A key aspect of the study highlighted in the manuscript is the rigorous daily monitoring of changes in bowel gas and analyzing how these changes affect the quality of the treatment plan. Additionally, the manuscript investigates the potential inaccuracies in the proton range caused by varying gantry beam angles. It aims to pinpoint the most advantageous gantry angles that would reduce deviations from the intended treatment plan, thereby improving the safety and effectiveness of proton therapy for these young patients.

**Abstract:**

(1) Background: Proton therapy, a precise form of radiation treatment, can be significantly affected by variations in bowel content. The purpose was to identify the most beneficial gantry angles that minimize deviations from the treatment plan quality, thus enhancing the safety and efficacy of proton therapy for Wilms’ tumor patients. (2) Methods: Thirteen patients with Wilms’ tumor, enrolled in the SJWT21 clinical trial, underwent proton therapy. The variations in bowel gas were systematically monitored using daily Cone Beam Computed Tomography (CBCT) imaging. Air cavities identified in daily CBCT images were analyzed to construct daily verification plans and measure water equivalent path length (WEPL) changes. A worst-case scenario simulation was conducted to identify the safest beam angles. (3) Results: The study revealed a maximum decrease in target dose (ΔD100%) of 8.0%, which corresponded to a WEPL variation (ΔWEPL) of 11.3 mm. The average reduction in target dose, denoted as mean ΔD100%, was found to be 2.8%, with a standard deviation (SD) of 3.2%. The mean ΔWEPL was observed as 3.3 mm, with an SD of 2.7 mm. The worst-case scenario analysis suggested that gantry beam angles oriented toward the patient’s right and posterior aspects from 110° to 310° were associated with minimized WEPL discrepancies. (4) Conclusions: This study comprehensively evaluated the influence of bowel gas variability on treatment plan accuracy and proton range uncertainties in pediatric proton therapy for Wilms’ tumor.

## 1. Introduction

Wilms’ tumor, also known as nephroblastoma, is the most common childhood renal neoplasm. The history of Wilms’ tumor treatment is marked by the integration of surgery, radiation therapy, and chemotherapy, leading to significant improvements in survival rates. Early efforts combined surgery and radiotherapy to achieve a 47% survival rate by 1950. The addition of chemotherapy, specifically an antibiotic called dactinomycin in 1966 boosted the survival rate to 80% [1]. The National Wilms’ Tumor Study Group (NWTSG), organized in 1968, achieved survival rates above 90% for non-anaplastic diseases by the 1990s through the refinement of chemotherapy and radiotherapy [2,3,4]. NWTSG recommended a radiation dose of 10.8 Gy to the abdomen, usually given as 1.8 Gy × 6 daily fractions [5].

Since the first studies conducted for the management of Wilms’ tumor, one of the goals has been the selection of the correct intensity of therapy based on histology and stage. Over the years, radiation doses have been reduced, but the volumes of treatment, flank, whole abdomen, and whole lung irradiation have remained relatively unchanged. Only recently there has been limited application of modern radiation therapy techniques and modern target volumes, such as intensity modulated radiation therapy (IMRT), volumetric modulated arc therapy (VMAT), or intensity modulated proton therapy (IMPT), to this patient population [6,7]. These techniques have the potential to impose reduced exposures to adjacent non-involved tissues in the abdomen including the bowel, liver, kidney, and spine, which may reduce common radiation-related late effects seen in this population [8].

Among the above-noted techniques being investigated, proton therapy has the greatest potential to reduce adjacent normal tissue exposures, but it encounters challenges when dealing with beam path heterogeneities and daily variabilities including tissue discrepancies, bowel gas variation, and respiratory motion. These unexpected variations along the proton beam path can significantly affect treatment accuracy and plan quality [9,10]. Variations in bowel filling, either with solid, liquid, or gas, can substantially alter the path of a proton beam, resulting in inaccuracies in the range of the proton therapy and changes to the dose distribution [11,12]. The impact of changes in bowel density does not appear to be restricted to the adult population and has been noted in studies of pediatric patients undergoing proton therapy for abdominal disease sites [13,14]. Proton therapy of targets in the abdomen and pelvis, particularly using IMPT, showed significantly worse D95% target coverage compared to photon-based techniques and was also highly impacted by the beam angle and the tissues along the beam path [14,15]. This highlights the need to carefully select a gantry angle that reduces the impact of bowel density changes and to also frequently monitor air pockets along the beam path through regular image guidance and execute adaptive re-planning as necessary.

We are currently conducting a prospective clinical trial testing the potential of proton therapy, with the modern conformal flank target, to reduce the developmental and end-organ late effects seen following the use of abdominal radiation in children. The SJWT21 trial (A Reduced Surgical and Radiation Therapy Treatment Paradigm Incorporating Proton Beam for Patients with Favorable Histology Wilms’ Tumor) treats a limited conformal target in the retroperitoneal space utilizing protons for patients historically requiring flank or renal irradiation in the treatment of stage III-V Wilms’ tumor [16]. Since the activation of the study, we have selected proton therapy beam angles to treat the patients who were enrolled based on prior experience and in-house planning studies. This manuscript describes the proton therapy for the initial 13 patients who underwent abdominal irradiation following nephrectomy or partial nephrectomy. We analyzed variations in bowel gas on a daily basis and their influence on the quality of the treatment plan. We investigated the potential errors in the proton range associated with different gantry beam angles in the abdomen and specifically sought to identify gantry beam angles that would minimize the risk of deviations from the planned treatment quality, thereby enhancing the safety and effectiveness of proton therapy for children with Wilms’ tumor.

This paradigm shift from conventional 2D planning to proton therapy in the treatment of pediatric Wilms’ tumor underscores the ongoing efforts to improve therapeutic outcomes while minimizing long-term side effects, particularly crucial in a pediatric setting. Future research and clinical trials are expected to continue to refine these approaches, focusing on maximizing therapeutic efficacy while safeguarding the well-being of young patients.

## 2. Materials and Methods

### 2.1. Patient Selection and Image Data

Thirteen Wilms tumor patients who enrolled in our prospective clinical trial (SJWT21 [16]) with favorable histology Wilms’ tumor were treated with proton therapy to the abdomen for either a modified flank/kidney field or liver irradiation.

The imaging data were acquired on the 13 patients using a Philips Vereos PET/CT system (Philips Healthcare, Cleveland, OH) with the clinical helical scan protocol of chest (120 kVp, 500 mm field of view (FOV), 512 × 512 matrix size, and slice thickness of 1.5 mm) for free-breathing scan. 4D CT imaging was performed by using the same Philips Vereos PET/CT scanner with 120 kVp, 0.06 spiral pitch factor, 0.625 mm collimation, a 500 mm FOV, and a slice thickness of 3 mm. The corresponding respiratory cycles were captured via pneumatic bellows belt signals. The number of 10 respiratory phases was sampled by the phase-binning technique for 4D CT reconstruction. A 4D CT scan was used to determine the full excursion of CTV and delineate internal target volume (ITV), if necessary. ITV was drawn if the internal target motion exceeded 3 mm as observed from a 4D CT scan. Free-breathing scan was used for proton dose calculation in Eclipse treatment planning system (TPS) (Varian Medical Systems Inc., Palo Alto, CA, USA).

### 2.2. Treatment Planning Scheme and Daily CBCT Analysis for Dose Accumulation

Target volumes were defined based on the pathologic findings. For patients with multiple pathologic findings as indications for radiotherapy (e.g., involved lymph nodes and surgical margin), the target volumes were generated per SJWT21 protocol. The name and the definition of target volumes for Wilms’ tumor used in TPS were described as follows:

- GTV_P: Patients with unresected gross tumor volume (GTV) at the primary (P) site
- CTV_P: Patients with local spill or evidence of rupture contained to the flank, or patients with an involved surgical margin after upfront surgical resection or delayed surgical resection, or patients with tumor thrombus removed in more than one piece or incompletely resected including 5 mm margin over GTV_P
- GTV_LN: Patients with unresected gross lymph node disease
- CTV_LN: Patients with para-aortic, para caval, and renal hilar lymph node (LN) involvement including 5 mm margin over GTV_LN
- GTV_BWT: Patients with cut surgical surface within the residual kidney as involved with microscopic bilateral Wilms’ tumor (BWT)
- CTV_BWT: Patients with an involved surgical margin in the partial nephrectomy specimen including a 5 mm margin over GTV_BWT
- ITV_P, ITV_LN, ITV_BWT: Internal motion to expand CTV_P, CTV_LN, CTV_BWT based on 4D CT study

In the proton treatment planning, a constant relative biological effectiveness (RBE) of 1.1 was used to account for the increased effectiveness of protons in destroying cells compared to photons. Dose prescriptions varied according to the disease stage and surgical findings in the planning. They were either administered as 10.8 GyRBE with 1.8 GyRBE across 6 daily fractions or 10.5 GyRBE with 1.5 GyRBE over 7 daily fractions, as determined within the treatment planning system. The goal for target coverage in the treatment plan was to ensure that 100% of the target volume receives the full 100% of the prescribed dose, which is expressed as D100% equals 100%. Constraints were set for normal structures including contralateral kidney and liver as organs-at-risk (OARs). Specifically, it was required that 50% of OAR volumes receive a dose less than 5 GyRBE. Setup uncertainty of 3 mm or 5 mm and range uncertainty of 3% or 5% were utilized in the robust plan optimization. The choice of either 3 mm/3% or 5 mm/5% robustness optimization parameters was decided by the physician based on multiple factors such as the predictions of setup reproducibility, extent of tumor motion, proximity to OARs, bowel gas variations, and beam angle selections. Table 1 outlines the patient demographics with disease sites, beam orientations, target volumes, prescriptions, and robust plan optimization parameters.

In the original planning, the presence of bowel gas or air along the path of the proton beam was identified using an image thresholding tool, with a range set between −500 Hounsfield Units (HU) and −1000 HU in Eclipse TPS. These areas were then delineated and overridden to match the densities of surrounding tissues, specifically between 20 HU and 40 HU, as observed in the planning CT scans. This planning workflow was implemented to ensure a uniform dose distribution across the target within the beam path.

The process of monitoring bowel gas variations involved the systematic use of daily Cone Beam Computed Tomography (CBCT) scans. For each patient, depending on the prescription of either 6 or 7 fractions, a total of 81 CBCT scans from 13 patients were meticulously analyzed. These CBCT images were then imported into the Eclipse TPS. Within this system, the same image thresholding technique was utilized, focusing on the detection of air pockets in the abdominal area, specifically in regions registering between −500 HU and −1000 HU. Once these air pockets were identified in the daily CBCT images, they were contoured and transferred into the original planning CT scans. The transfer of daily air pockets was crucial for recalculating the daily dose distributions, known as the “plan of the day” or for constructing daily verification plans. After the completion of all treatment fractions, a thorough comparison and analysis were conducted. This involved accumulating all the daily verification plans and comparing them against the original planned parameters and dose volume histograms. This comparative analysis aimed to assess any deviations from the planned dose distribution and to understand the impact of bowel gas changes on the effectiveness and accuracy of the treatment.

### 2.3. The Worst-Case Scenario Simulation

The involuntary nature of peristaltic movement in the GI tract renders the task of precisely predicting the location and volume of bowel gas at any specific time quite formidable. This unpredictability is crucial in proton therapy, as gas voids within the GI tract can significantly alter the path and range of the proton beam, leading to potential inaccuracies in the treatment. To address this challenge, a meticulous worst-case scenario analysis was employed for each patient’s treatment planning. This analysis involved creating a simulated plan for each of the thirteen patients that encompassed a full 360° rotation around the patient, generating 36 distinct beam angles at 10° intervals and determined WEPL changes when the entire stomach and intestines were filled with air. This comprehensive simulation aimed to identify the safest beam entry points considering the variable nature of bowel gas distribution.

In the context of this simulation, two scenarios were considered for each patient. In the best-case scenario, any identified air pockets within the GI tract were overridden to match the densities of adjacent tissues, as outlined in Section 2.2. Conversely, in the worst-case scenario, a more extreme approach was taken. The entire stomach and intestines were delineated, and their densities were overridden to reflect an air density of −1000 HU in the original CT scans. This method allowed for an assessment of the maximum possible impact of bowel gas on proton therapy. Furthermore, for each field in each patient’s plan, the water equivalent path length (WEPL) was calculated. This calculation was crucial to estimate the extent of proton range shifts. The WEPL provided a critical metric for determining the distance the proton beam would traverse, equivalent to traveling through water, from the patient’s surface to the distal edge of the target volume, taking into account the presence of air pockets. The methodology for this calculation and its implications were elaborated in Section 2.4 of the study. Overall, this rigorous simulation process provided vital insights into the potential effects of bowel gas on proton beam therapy, allowing for more precise and effective treatment planning.

### 2.4. Water Equivalent Path Length Method

In this study, the WEPL method, pivotal for estimating the proton range within the body, was employed. A specialized MATLAB algorithm developed in-house (The MathWorks Inc., Natick, MA, USA), facilitated the WEPL calculations. The approach involved calculating the WEPL up to the distal surface of the patient’s target volume. This was achieved through linear integration of the Relative Proton Stopping Power (RPSP), guided by a stoichiometric calibration curve correlating CT number with RPSP, a standard in treatment planning [17]. Using this calibration curve, RPSP values were integrated linearly for each voxel from the planning CT scans. The integration followed the path of the beam’s eye view (BEV) from the external surface of the patient’s body to the distant surface of the target volumes [18,19]. This integration took into account any overridden structures along the path of the beam. For each field, WEPL histograms were generated, and the 90th percentile of these distributions was used to ascertain the estimated range of protons along the beam path. The study defined WEPL differences using a specific mathematical formulation, ΔWEPL, as described in Equation (1) used in the study of Section 2.2 and Equation (2) used in the study of Section 2.3.

(1) $${\Delta WEPL}_{patient\;cases}={WEPL}_{original\;plan}-{WEPL}_{daily\;plan},$$

(2) $${\Delta WEPL}_{simulation}={WEPL}_{without\;air}-{WEPL}_{with\;air},$$

## 3. Results

### 3.1. Impact of Daily Bowel Gas Changes on Plan Quality

The study systematically assessed dose accumulation in 13 patients with Wilms’ tumors, utilizing 81 CBCT images for daily evaluation. This analysis was juxtaposed with the original treatment planning parameters, focusing on both the target areas and OARs. Additionally, a comprehensive WEPL analysis was conducted, encompassing 188 evaluations across each treatment day and field, specifically considering the impact of bowel gas presence in the beam’s path. Key findings included a maximum observed decrease in the target dose (ΔD100%) of 8.0%, correlated with a WEPL variation (ΔWEPL) of 11.3 mm. The average dose reduction in the target, represented as mean ΔD100%, was 2.8% with a standard deviation (SD) of 3.2%. Moreover, the mean ΔWEPL recorded was 3.3 mm, with an SD of 2.7 mm. Statistical analysis revealed a significant correlation between the degradation of the treatment plan’s quality and the ΔWEPL, with a determined *p*-value of 0.01 under a significance level (α) of 0.05. This suggested a notable influence of daily proton range variations in terms of WEPL on the treatment plan qualities due to bowel gas changes. Table 2 presents a comparison of dose parameters between the original treatment plans and the accumulated dose plans, as well as the differences in WEPL for each field, comparing the original CT-based plans with the daily CBCT-based plans.

**Figure 1:**
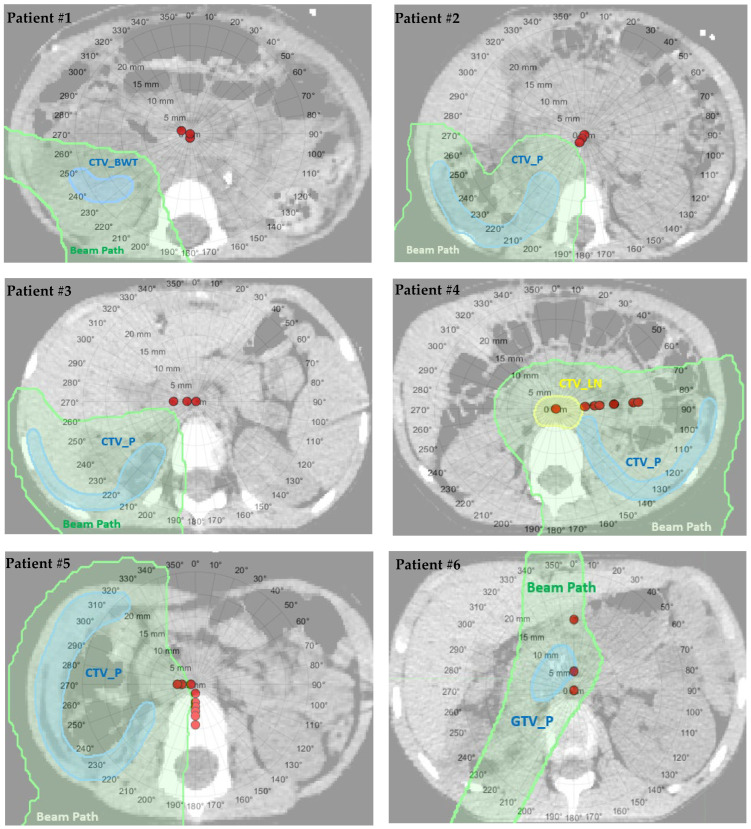

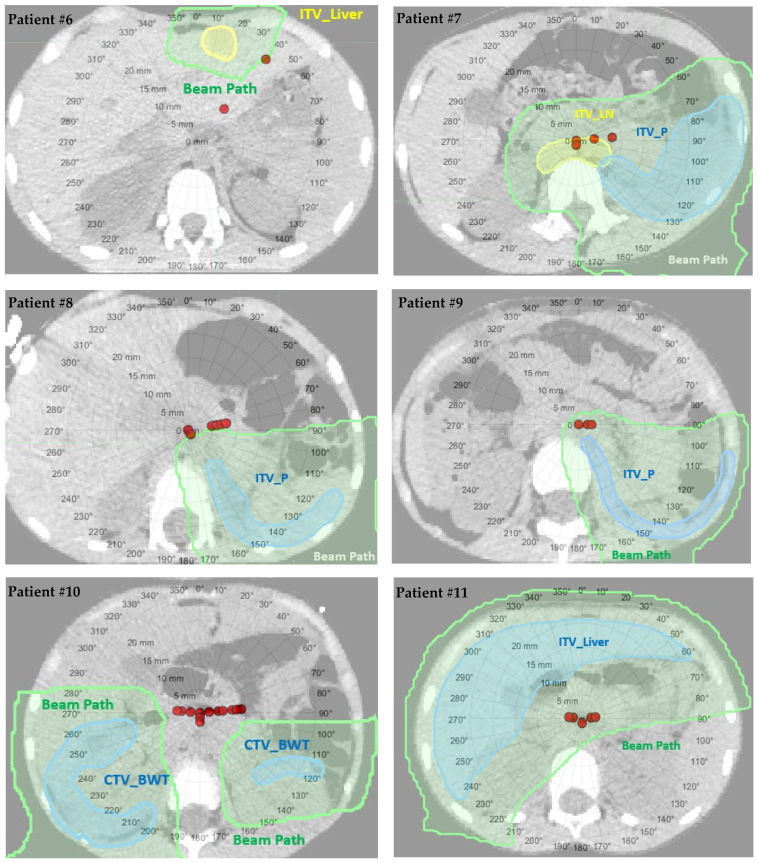

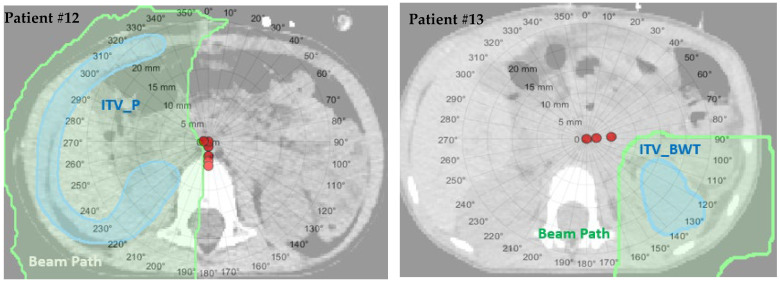
Polar plots, mapped in function of gantry angle and ΔWEPL (red data points), overlaid on axial CT images for thirteen Wilms’ tumor patients, highlighting the beam paths (20% isodose lines) in green shaded region and target volumes in blue and yellow.

The analysis revealed that the location of Wilms’ tumors and the selection of beam gantry angles were critical factors in treatment efficacy. Notably, left-sided tumors in patients #4, #6, #7, #8, and #10 exhibited significant reductions in target coverage, with decreases of 5.1%, 8.0%, 8.2%, 6.9%, and 5.5%, respectively. Conversely, tumors located on the right side showed a beneficial effect due to the proximity of the liver, the body’s second-largest organ, which mitigated the impact of bowel gas in the abdomen for patients #1, #2, #3, #5, #11, and #12. Tumors in the left retroperitoneal region (patients #9 and #13) were less affected by changes in bowel gas when compared to other left-sided tumors (patients #4, #6, #7, #8, and #10), especially when posterior or posterior oblique beams were utilized in the treatment. In terms of OARs, liver sparing adhered to clinical constraints for all patients except #5 and #12, where the liver was involved in the tumor volume. Additionally, sparing of the contralateral kidney was achieved in all cases except for patient #10, who had bilateral Wilms’ tumors involving a portion of the left kidney. Figure 1 showcases polar plots for patient cases, illustrating the gantry beam angles and corresponding WEPL variations between original and daily CBCT-based treatment plans.

This patient case study was limited by the number of gantry angles and associated beam paths utilized in the planning. This limitation necessitated a comprehensive simulation study, which involved using beam entrances at every 10° interval over a full 360° rotation. The findings and implications of this extended simulation were discussed in the subsequent Section 3.2.

### 3.2. The Worst-Case Scenario Simulation Results

The study conducted a worst-case scenario analysis by simulating treatment plans for thirteen patients. Each plan included a comprehensive 360° rotational arc around the patient, creating 36 unique beam angles at intervals of 10°. The worst-case scenario simulation was designed to assess changes in ΔWEPL under the condition that the patient’s stomach and intestines were completely filled with air. A total of 468 ΔWEPL measurements were generated, correlating with the various beam entry angles. Statistical analysis revealed that the median ΔWEPL across all gantry angles was 5 mm, with an SD of 17.6 mm. Upon examination of the beam entries, stark differences in ΔWEPL were observed across various beam orientations. The posterior beam angles indicated a median ΔWEPL of 1 mm (SD 9 mm), while the anterior beam angles displayed a median ΔWEPL of 11 mm (SD 20.5 mm). In contrast, the right beam angles revealed a median ΔWEPL of 2 mm (SD 15.3 mm), and the left beam angles showed a median ΔWEPL of 8 mm (SD 18.5 mm). 

**Figure 2:**
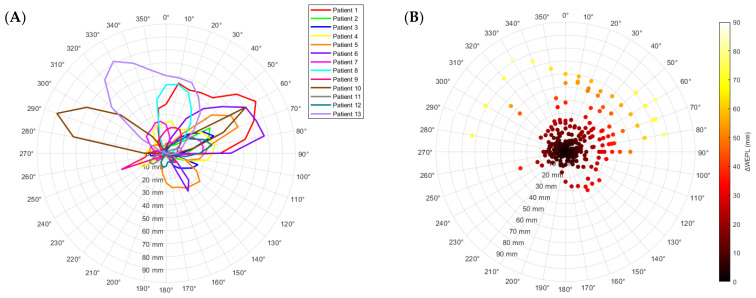
Polar plots of ΔWEPL variations as a function of gantry angle per patient (**A**), and heat map (**B**).

The results of the study demonstrated that in scenarios where the abdominal area was filled with air, the gantry beam angles directed towards the patient’s right and posterior sides, notably the right posterior quadrant, presented reduced discrepancies in WEPL. This underscored the strategic significance of these particular beam angles in effectively minimizing WEPL inaccuracies in such scenarios. Figure 2 illustrates the variations in WEPL through two distinct representations: a polar plot highlighting each patient (Figure 2A) and a polar heat map emphasizing the data cluster (Figure 2B), both depicting the relationship between WEPL differences and gantry angles.

**Figure 3:**
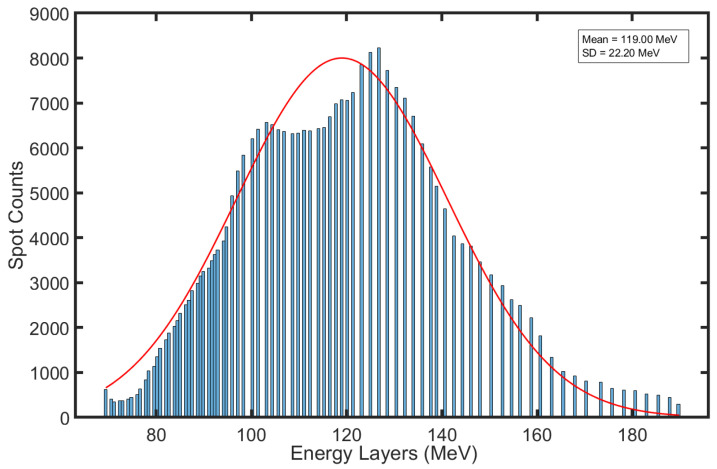
Energy histogram of thirteen Wilms’ tumor patients extracted from the original treatment plans.

To delve deeper into the effect of proton range uncertainty on the plan quality, the histogram of energy layers for the thirteen patients was plotted. The findings, as illustrated in Figure 3, indicated that the total counts of spots and energy layers in these treatment plans were 301,349 and 1852, respectively. A Gaussian fit applied to the histogram data revealed an average energy layer of 119 MeV, correlating to a 105 mm range in water, which was deduced from the treatment plans. Furthermore, it can be inferred that a 3% range error at a 100 mm range would result in a WEPL error of approximately 3 mm. The data presented in Table 1 and Table 2 showcased that the patients #1, #2, #3, #9, #11, and #13, who had a ΔWEPL error of ≤3 mm, achieved a dose reduction of only ≤1.5% in the target D100%, under treatment plans optimized with 3% proton range error. Conversely, patients #7, #8, and #10, despite being subject to the same 3% range uncertainty robust optimization, experienced a more substantial dose discrepancy ranging between 5.5% and 8.2% in target D100% due to ΔWEPL errors having >3 mm. These findings underlined the critical nature of selecting appropriate proton range uncertainty parameters in the robust plan optimization, hence, emphasizing the necessity of tailoring these parameters based on the depth of the target volume to be treated.

**Figure 4:**
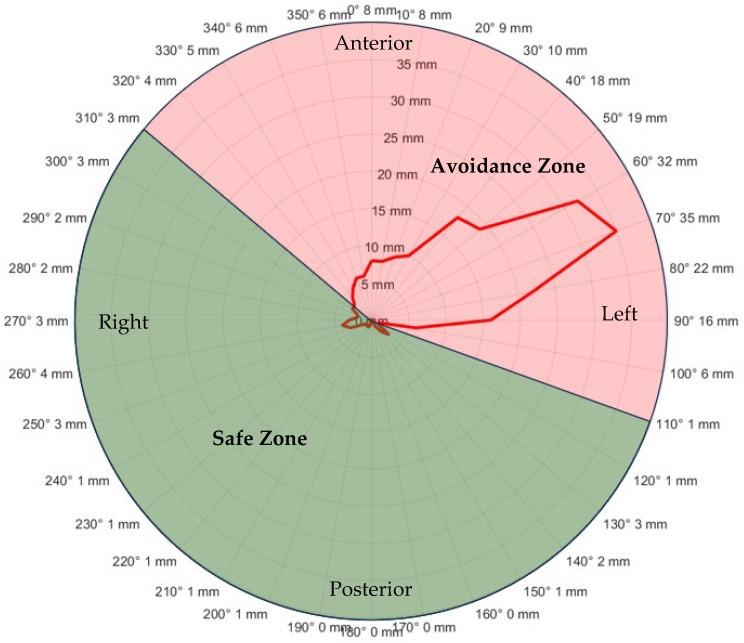
Median ΔWEPL values in function of gantry beam angles based on the worst-case scenario simulation, showing safe (green-shaded) and avoidance (red-shaded) zones.

Utilizing the benchmark data from thirteen Wilms’ tumor patients, the simulation provided an in-depth overview of how gantry angle correlated with WEPL errors at each 10° interval. A polar plot, depicted in Figure 4, was created to illustrate the median ΔWEPL for every 10° interval of the gantry angle. This plot highlighted zones of avoidance and safety for gantry beam angles, informed by the average energy layer data derived from thirteen Wilms’ tumor patients and a 3% range (or 3 mm for 100 mm range in water) error margin. These insights were proposed for scenario-based optimization in the treatment planning as proactive measures when designing the treatment planning.

## 4. Discussion

In the field of proton therapy, precise beam angle selection and understanding of WEPL variations are critical for optimizing treatment plan quality. These factors can significantly impact dose distribution and the sparing of healthy tissues. Numerous research groups investigated how daily variations in WEPL affect the efficacy of treatment plans, proposing methods to address daily proton range discrepancies. One specific study [20] focused on the robustness of incident proton beam angles against daily anatomical changes in patients with lung cancer. The results of this study highlighted a substantial patient-to-patient variation in WEPL relative to the beam angle, with observed changes in WEPL (median: 3.3 mm, range: 0.0–41.1 mm). The study indicated that even minor changes in the beam angle could result in significant variations in WEPL and have a considerable impact on the dose distributions. In another study [21] evaluating lung cancer patients, the authors found that breathing-induced motion could cause substantial WEPL variations for different beam angles. The study noted a strong correlation (correlation coefficients ranging between 0.92 and 0.98) between WEPL changes and reduction in the target dose coverage, emphasizing the sensitivity of dose distribution to WEPL variations induced by respiratory motion. A research team [22] developed a method for identifying patient-specific robust proton beam angles for lung tumor irradiation. The approach was the use of ray tracing to estimate WEPL maps or baselines for the ITV at every 5° gantry interval in the axial plane. These maps were then used to assess how shifts in the planning CT impact the WEPL, and consequently, the dose delivered to the target.

While many studies focus on beam angle selection and WEPL in lung cancer, research on other body cases is less common. Another study [23] explored the impact of anatomical and setup changes on proton range variation in head and neck cancer treatment. Utilizing planning CT and weekly CBCT images, the study estimated the proton range through WEPL calculations. Notably, it found the largest median and quantile values of distal WEPL difference at posterior oblique angles, highlighting a greater risk of overdosing surrounding normal tissues. A research team [24] developed a method to evaluate the robustness of proton therapy plans for pelvic lymph node irradiation, analyzing the impact of inter-fractional motion. This involved optimizing proton plans for specific lymph node targets and assessing variations in WEPL. The study also compared CTV margins, suggesting that a 7 mm margin could be sufficient for some patients in managing inter-fractional movement. The 5 mm margins also showed good target coverage, except for the most anterior angles. For anterior angles, bladder filling and lymph node shape were found to significantly influence the proton range.

In this study, we conducted a comprehensive analysis of thirteen patients diagnosed with Wilms’ tumor, treated in the SJWT21 clinical trial. The primary focus of this study was to evaluate the influence of bowel gas on the quality of treatment plans. We identified a statistically significant correlation (*p*-value = 0.01, α = 0.05) between the variation in WEPL and the degradation of plan quality, particularly concerning the D100% target metric (Section 3.1). This finding underscored the critical impact of WEPL variations on treatment efficacy in Wilms’ tumor cases. The limitation of this study, however, lies in the particular beam angles selected for the original treatment planning. 

To address this limitation and enhance the robustness of our findings, we conducted an extensive worst-case scenario simulation (Section 3.2), incorporating a full 360° rotation of beam angles at intervals of 10°. This approach allowed for a more comprehensive understanding of the influence of beam angle variation on treatment plan quality. The simulation study revealed that the posterior and right beam angles demonstrated smaller median changes in ΔWEPL, measuring 1 mm and 2 mm, respectively. This finding indicated that beam angles directed towards the right posterior quadrant could be potentially more favorable for treatment in the simulation of thirteen patients. 

A key aspect of the study was the investigation of the relationship between selected gantry angles and the resultant WEPL errors, particularly those arising from changes in bowel gas. A vital outcome of this research involved determining the extent of WEPL error that could be tolerated within the treatment plan, along with exploring potential proactive strategies for mitigating proton range errors in the treatment planning. In future directions, an automated algorithm could be devised to predict the safe beam angles based on the simulation CT and the location of tumor volumes to be used in the treatment planning of the prospective Wilms’ tumor patients.

## 5. Conclusions

This research focused on evaluating the impact of bowel gas variability on the precision of treatment plans and the associated proton range uncertainties in pediatric proton therapy for Wilms’ tumor. A key objective was to determine which gantry beam angles would most effectively reduce the likelihood of deviation from the intended treatment quality. This is crucial for enhancing both the safety and efficacy of proton therapy in treating children with Wilms’ tumor. Our method involved a detailed simulation and analysis, utilizing data from thirteen pediatric patients. This process enabled us to uncover significant correlations between the angles of the gantry beams and the errors in WEPL. The comprehensive analysis of this study suggested that gantry beam angles oriented towards the patient’s right and posterior aspects from 110° to 310°, were associated with minimized WEPL discrepancies and could be potentially more favorable for treatments of Wilms’ tumor patients. The insights gained from this study are not only critical for improving treatment approaches for Wilms’ tumor but also hold potential applicability in other conditions where bowel gas variation affects proton abdominal irradiation.

## Figures and Tables

**Table 1:** Summary of patient characteristics. Abbreviations: LPO, left posterior oblique; RPO, right posterior oblique; AP, anterior-posterior; RL, right lateral; PA, posterior-anterior; LL, left lateral; RAO, right anterior oblique; LAO, left anterior oblique. M, male; F, female. R, right; L, left.

Patient Number	Patient Age (Years)	Sex	Disease Site	Beam Arrangements	Target Volume	Prescription Dose (GyRBE)	Setup (mm)/Range (%) Uncertainty in Original Plan
1	2	M	R Kidney	RAO, LPO, PA	CTV_BWT	10.8	3/3
2	1	F	R Abdomen	RPO, PA	CTV_P	10.8	3/3
3	5	M	R Abdomen	RL, PA	CTV_P	10.5	3/3
4	3	M	L Abdomen	PA, LAO	CTV_P + CTV_LN	10.5	5/5
5	4	F	Partial Liver	PA, RL	CTV_P	10.5	5/5
6	13	M	L/R Abdomen	AP, RPO, LAO	ITV_Liver + GTV_P	10.8	5/5
7	9	F	L Abdomen	PA, LAO	ITV_P + ITV_LN	10.8	3/3
8	4	F	L Abdomen	LPO, LAO	ITV_P	10.8	3/3
9	5	F	L Abdomen	LPO, LL	ITV_P	10.8	3/3
10	5	M	L/R Partial Kidney	PA, RAO, LAO	CTV_BWT	10.5	3/3
11	6	F	Liver	PA, RAO, LAO	ITV_Liver	10.5	3/3
12	4	M	Partial Liver	PA, RAO	ITV_P	10.8	5/5
13	4	F	L Kidney	PA, LAO	ITV_BWT	10.8	3/3

**Table 2:** Summary of treatment plan and dose–volume parameters. Abbreviations: D100%, 100% of the target volume of interest receives a percentage of prescription dose; D50%, 50% of the OAR volume receives a dose in GyRBE; ΔWEPL, the difference of WEPL for each field between original CT and daily CBCT-based plans. N/A*, there is no contralateral kidney; N/A**, and liver is the target.

Patient Number	Target D100% in Original Plan (%)	Target D100% in Daily Accumulation (%)	Contralateral Kidney D50% in Original Plan (GyRBE)	Contralateral Kidney D50% in Daily Accumulation (GyRBE)	Liver D50% in Original Plan (GyRBE)	Liver D50% in Daily Accumulation (GyRBE)	Target ΔD100% (%)	ΔWEPL Mean ± SD (mm)
1	98.9	98.4	N/A*	N/A*	0.1	0.1	0.5	1.0 ± 0.8
2	99.6	99.6	0.0	0.0	0.0	0.0	0.0	0.8 ± 0.9
3	97.0	97.0	0.0	0.0	0.7	0.7	0.0	1.9 ± 2.2
4	97.9	92.8	0.1	0.1	0.6	0.7	5.1	5.7 ± 6.5
5	99.6	99.5	0.0	0.0	8.2	8.2	0.1	3.9 ± 2.3
6	99.5	91.5	0.0	0.0	0.0	0.0	8.0	11.3 ± 9.6
7	97.0	88.8	0.2	0.3	0.0	0.0	8.2	3.4 ± 3.2
8	99.0	92.1	0.0	0.0	0.0	0.0	6.9	3.4 ± 3.0
9	97.6	96.1	0.0	0.0	0.0	0.0	1.5	1.3 ± 1.4
10	98.6	93.1	7.4	7.4	0.0	0.0	5.5	3.8 ± 2.5
11	85.4	85.0	0.1	0.1	N/A**	N/A**	0.4	2.0 ± 0.8
12	87.4	87.3	0.0	0.0	8.5	8.6	0.1	1.7 ± 1.7
13	99.0	98.8	0.0	0.0	0.0	0.0	0.2	2.3 ± 2.5

## Data Availability

The data that support the findings of this study are available from the corresponding author, upon reasonable request.
